# GraphDNA: a Java program for graphical display of DNA composition analyses

**DOI:** 10.1186/1471-2105-8-21

**Published:** 2007-01-23

**Authors:** Jamie M Thomas, Daniel Horspool, Gordon Brown, Vasily Tcherepanov, Chris Upton

**Affiliations:** 1 Department of Microbiology and Biochemistry, University of Victoria, Victoria, BC, V8W 3P6, Canada

## Abstract

**Background:**

Under conditions of no strand bias the number of Gs is equal to that of Cs for each DNA strand; similarly, the total number of Ts is equal to that of As. However, within each strand there are considerable local deviations from the A = T and G = C equality. These asymmetries in nucleotide composition have been extensively analyzed in prokaryotic and eukaryotic genomes and related to chromosome organization, transcription orientation and other processes in certain organisms. To carry out analysis of intra-strand nucleotide distribution several graphical methods have been developed.

**Results:**

GraphDNA is a new Java application that provides a simple, user-friendly interface for the visualization of DNA nucleotide composition. The program accepts GenBank, EMBL and FASTA files as an input, and it displays multiple DNA nucleotide composition graphs (*skews *and *walks*) in a single window to allow direct comparisons between the sequences. We illustrate the use of DNA skews for characterization of poxvirus and coronavirus genomes.

**Conclusion:**

GraphDNA is a platform-independent, Open Source, tool for the analysis of nucleotide trends in DNA sequences. Multiple sequence formats can be read and multiple sequences may be plotted in a single results window.

## Background

The nucleotide composition of genomic DNA is very variable. This variation is not limited to non-coding DNA or influencing codon-usage, it also results in changes to the amino acid composition of proteins. For example, the redundancy of the genetic code does not completely buffer the amino acid composition of orthologous proteins from Vaccinia virus (VACV; an *Orthopoxvirus*; ~65% A+T) and Molluscum contagiosum virus (MOCV; a *Molluscipoxvirus*; 35% A+T). The amino acid composition of the DNA polymerases of VACV and MOCV illustrate that the propensity for certain amino acids to be more common in proteins encoded by A+T or G+C rich genes; the VACV and MOCV proteins contain the following percentages of lysine (7.1, 2.9), arginine (5.9, 9.6), alanine (4.0, 9.5), leucine (9.3, 12.2) and isoleucine (7.4, 3.2), respectively.

There is also considerable variation in DNA composition within a genome. Regions rich in repeated DNA often have a nucleotide composition quite different to the genome average. Similarly, all poxvirus promoters are very A+T rich regardless of the average genome nucleotide composition. However, it is less well appreciated that there is also a wide variation in the DNA composition of individual genes, the A+T% range for individual genes in VACV and MOCV is 55.1 – 72.3 and 24.0 – 51.8, respectively. The driving force for divergence in DNA composition among evolutionary related poxviruses is unknown, as is the ancestral composition, but range of DNA composition among genes is determined by at least 2 components: 1) the natural resistance of some genes to change because of restrictive amino acid requirements, and 2) the acquisition of novel genes after speciation of the viruses.

Other processes related to DNA replication, repair and transcription may also influence nucleotide biases of the two DNA strands [1-5]. For example, since the coding strand of bacterial genomic DNA tends to be purine rich [6] and majority of genes are transcribed in the same direction as the movement of the replication fork there is asymmetric nucleotide composition along the genome such that the DNA composition may be used to predict the origin and termini of replication [7-9].

Genomic variations in nucleotide composition have been successfully studied using several graph-based methods. One such technique is to draw a cumulative skew diagram, which plots the relative amounts of two nucleotides in a given DNA sequence [10]. A GC skew, for example, is calculated across a genome as the sum of a series of sliding windows of specified length; the window size can be 1 or much larger for a complete genome. The shape of the resulting cumulative dinucleotide curves have been correlated with important sequence features including viral origins of replication [11] and genome rearrangements in bacteria [12]. The "DNA walk" is another method used to study nucleotide distribution, first described by Lobry [7,13] and used to detect origins of DNA replication in bacteria genomes. To graph a DNA walk, a direction (North, South, East, and West) is assigned to each of the four nucleotides and the sequence is then plotted on a graph, beginning at (0,0) and moving one step in the direction specified by each successive nucleotide.

This paper describes GraphDNA, a new Java software application, that was developed as a platform-independent application to present DNA skews and walks of multiple sequences in a single graph and thus assist in the comparative analysis of nucleotide composition asymmetry.

## Implementation

To support cross-platform utility, GraphDNA was developed as a Java application. Users initially access and launch the application from a web page using Java Web Start, which also automatically downloads updated versions of the program as they become available. This ensures users are taking advantage of improvements or added features in the latest software versions.

GraphDNA can read DNA sequences from several sources. First, this input can consist of a single file containing one or more DNA sequences or a series of separate sequence files can be loaded; the nucleotide sequence data may be in FASTA, EMBL or GenBank formats. Second, the application can connect to our VOCs database at the Viral Bioinformatics Resource Centre (VBRC) [14] that stores the complete genomes from more than 10 families of viruses; in this case, GraphDNA also receives the gene annotations for the genomes. GraphDNA has been successfully tested with a 20 Mb DNA sequence and can therefore handle all currently sequenced viral and bacterial genomes.

The time required by GraphDNA to load DNA sequences and display plots is dependent upon the size and number of sequences as well as the nucleotide window size used for calculating skews and walks. The time to load and display a GC skew for 4 poxvirus genomes (average size 200 Kb) was approximately 40 s with a default window size of 1 nucleotide; however, replotting the same data with a window size of 10 nucleotides took <5 s (2 GHz dual processor). When large (>200 Kb) DNA sequences are loaded, the program automatically selects a window size of 70 nucleotides and permits the user to change that window size, this reduces the processing and display times; for example, the time required for loading and plotting the GC skews of two 20 Mb DNA sequences was approximately 2 minutes on a dual 2 GHz desktop computer; replotting this data with another nucleotide skew took <45 s.

Additional features include: 1) the application can accept gapped multiple alignment files in FASTA format; this permits homologous regions of genomes to be kept in register and allows easier comparison of skews among several sequences. Horizontal lines are drawn at gapped positions; this reflects no change in nucleotide skew, but an increase in nucleotide count; 2) gene annotations are displayed underneath the main window if they have been loaded with the DNA sequences (transcript direction is shown and the gene name is also displayed when space permits), if multiple sequences have annotations, a menu allows the user to choose which annotations to display; 3) it is possible to zoom into regions of the plots (a selection is made by holding down the left mouse-button and dragging across the region), both the plot and the genome annotation displays are affected by this procedure and clicking the "*Recenter button*" switches back to the full genome view.

GraphDNA allows plots to be saved as PNG formatted files (*save as*, under *File *menu), the best resolution is obtained if the application window is stretched to its maximum size before saving; alternatively, screen images can be captured.

### Algorithms

In a "DNA walk" graph, sequences are plotted starting at X = 0, Y = 0. For each nucleotide (from position 1 in the sequence to the end), the position of the next point in the plot is calculated relative to the current position: for nucleotide C, G, T or A, the position moves north, south, east or west, respectively. If the current symbol is degenerate or a gap symbol, the position is unchanged. For a window size of k, every k'th point is actually drawn on the graph (but the calculations still include every nucleotide). A slider bar moves markers along the plots to locate specific regions of the sequences since the position of a particular nucleotide is solely dependent on the composition of the preceding nucleotide sequence, not on the position in the sequence.

Purine skews are calculated from the first position in the sequence to the last: for each nucleotide, increment a counter if this nucleotide is a purine; decrement it if it is a pyrimidine. The effect is to compute the number of purines minus the number of pyrimidines from the first position to the current one. The X-axis of the skew graph is the position in the sequence; the Y-axis is the value of the counter at this position. Keto and dinucleotide skews are calculated analogously, with the obvious differences. For a window size of k, every k'th position is drawn.

### Comparison to other programs

Several other programs have been written to display DNA strand composition asymmetries. DNASkew [15] is a Perl script limited to a command line interface; GenSkew [16] and Artemis [17] are Java applications, but are limited to certain nucleotide skews and cannot plot multiple sequences; Genometrician's Scooter™ [18] is able to perform DNA walks and skews, but is not Open Source or platform independent. GraphDNA was written to overcome these limitations and also to be compatible with the VOCs database [19] for access to viral genomes.

## Results and discussion

The GraphDNA application has three basic graphing functions: DNA walks, cumulative dinucleotide skews and cumulative purine/keto skews. The DNA walk analysis is most effective when nucleotide composition trends extend over a considerable length. For example, DNA walks have been successful in finding the origins and termini of replication in bacterial genomes [7,20].

**Figure 1:**
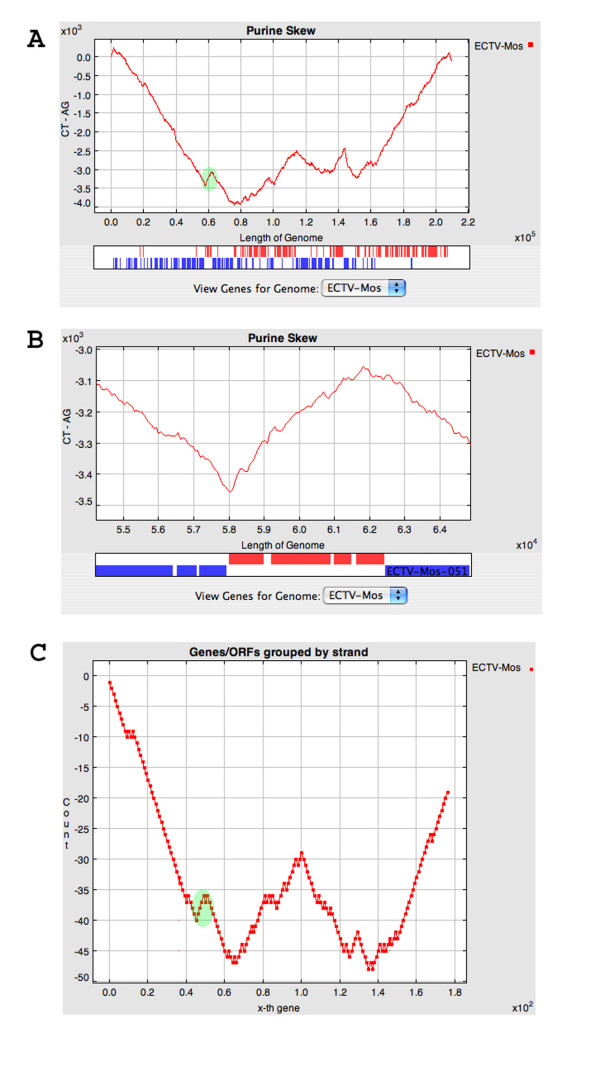
Panel A: Purine skew for the complete genome of Ectromelia virus strain Moscow (NC_004105.1). Gene annotations for this genome are show below the skew plot; genes are shown as red and blue bars to illustrate transcription towards the left and right ends of the genome, respectively. Green highlight shows 4 genes that are transcribed rightwards, which are flanked by genes transcribed leftwards. Panel B: Purine skew for the region highlighted in Panel A. Panel C: Plot of transcription orientation for Ectromelia virus genes. For genes transcribed to the left and right, the plot moves down and up, respectively. Green highlight as in Panel A.

An example of cumulative purine/keto skews is shown in Figure 1, which demonstrates that different regions of the ectromelia virus, an orthopoxvirus, genome have considerably different purine content. It was observed that the "W" shape of the purine skew (Figures 1A and 1B) was very similar to the pattern for transcriptional orientation of the viral genes along the linear genome (Figure 1C). Examination of the genes from multiple orthopoxviruses confirmed that the coding strand of poxvirus genes tends to be slightly purine rich [21]. This information can be used to help annotate novel genes in poxvirus genomes in that if there are small ORFs on both DNA strands, then the strand with the highest purine content is most likely to be the coding strand; however, purine content is only one of a variety of information sources used in the annotation process [22]. Cumulative skew analysis may also be pertinent to some eukaryotic DNA sequences; for example, the coding strand of *Leishmania major *strain Friedlin chromosome 1 [23] is associated with an excess of pyrimidines.

**Figure 2:**
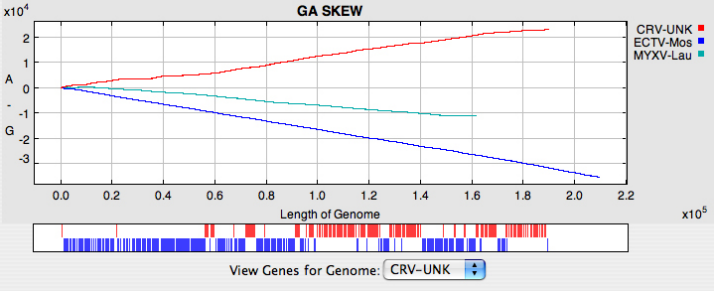
GA skews for the complete genomes of Ectromelia virus strain Moscow (NC_004105.1), Crocodilepox virus (NC_008030) and Myxoma virus (NC_001132.2); the genome sizes range from ~160 to 210 Kb. Gene positions and transcription direction (red – rightwards; blue – leftwards) are shown in a box underneath the plots; if space permits, gene names are also displayed. A *View Genes for Genome *menu is provided to allow the user to select between multiple genomes.

**Figure 3:**
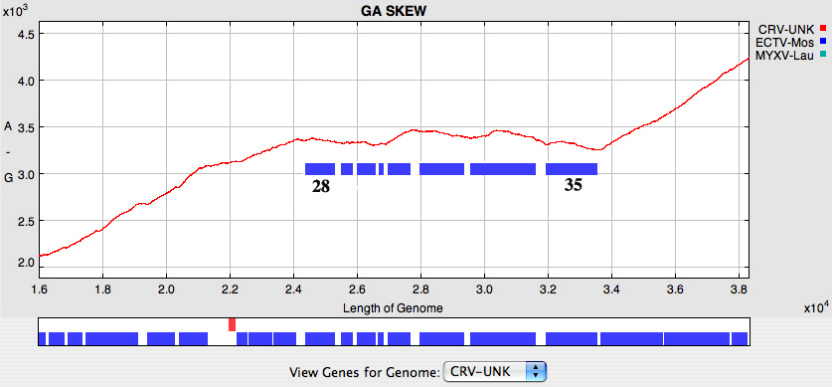
GA skew for the region 16 – 38 Kb of the Crocodilepox virus genome shown in Figure 2; note the change in scale shown. Blue bars on the plot represent genes 28, 29, 30, 31, 32, 33, 34 and 35.

The cumulative dinucleotide skews display the abundance of one nucleotide relative to another across the length of a DNA sequence that may represent a single gene or a complete genome. The program plots the cumulative dinucleotide counts calculated in a series of "windows", which may be as small as 1 nt, along the DNA sequences; GraphDNA offers all six possible cumulative skews (GC, AC, GT, TC, AG and AT). GC and AT skews have been widely used to predict termini and origins of replication in bacterial [1,9] and mammalian genomes [8], transcription start sites in plants and fungi [24], as well as transcription regions in the human genome [25]. In poxviruses, some skews, for example the GA skew, appear to be independent of the orientation of transcription, and instead reflect the overall nucleotide composition of the genome (Figure 2). However, when genomes are examined in more detail, heterogeneities can be observed. The GA skew of the 16 – 38 Kb region of the crocodilepox virus genome from Figure 2 is displayed in Figure 3. Genes 28, 29, 30, 31, 33, 34 and 35 appear to make up an island of unusual skew. This is interesting because genes 28, 33, 34 and 35 are distantly related and probably arose from ancient duplication events. Of all the genes in the region 28 – 35, only gene 30 has any similarity to other known proteins; it is similar to DNAJ-like molecular chaperones and has an ortholog in MOCV, another GC rich poxvirus. The unusual overall base composition of this region may be the result of incorporation of foreign DNA into an ancestral crocodilepox virus genome. It is likely that this was through multiple events and an early smaller DNA island may have served as an integration site for subsequent insertions.

**Figure 4:**
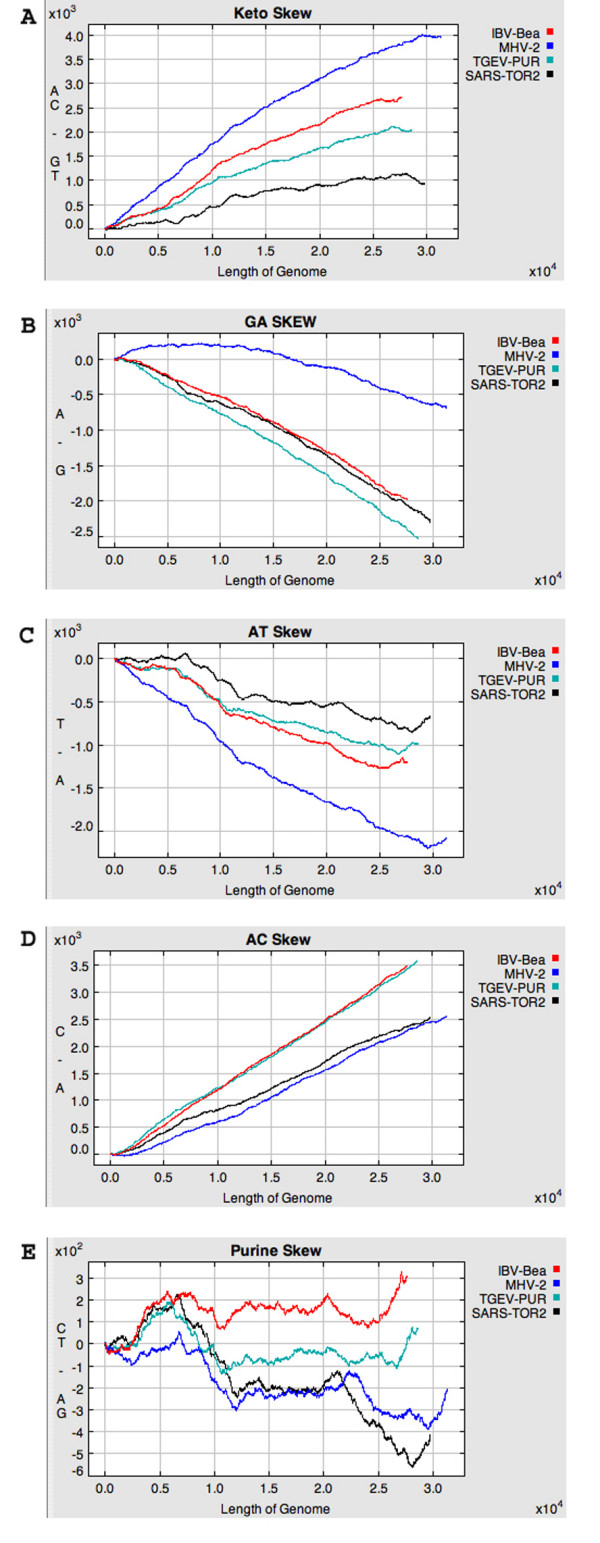
Panels A, B, C, D and E show keto, GA, AT, AC and purine skews for Transmissible gastroenteritis virus strain Purdue (Group 1; NC_002306), Murine hepatitis virus strain 2 (Group 2; AF201929), Avian infectious bronchitis virus strain Beaudette (Group 3; NC_001451) and SARS coronavirus strain Tor2 (NC_004718), respectively.

Since the above analysis of crocodilepox genes indicated that DNA composition signature can reflect evolutionary history even when protein sequences have diverged so far that reliable alignments are very difficult to generate and are limited to a very few essential amino acids (alignment of crocodilepox genes 28, 33, 34 and 35, results not shown), we looked at the relationship of SARS-coronavirus to other coronaviruses using nucleotide skews. When the SARS-coronavirus was first sequenced, it was reported that this novel virus was similarly and very distantly related to the three previously known groups of coronaviruses [26]. Subsequently, it was reported that SARS-coronavirus is slightly closer to the Group 2 coronaviruses [27,28] and that it probably arose through a series of recombination events [29]. However, an examination of composition skews revealed that for most skews SARS-coronavirus and mouse hepatitis virus (MHV), a Group 2 coronavirus are the most different; for example, see the keto, GA and AT skews in Figure 4A, 4B and 4C, respectively. Only the AC skews are most similar for SARS-coronavirus and the Group 2 MHV (Figure 4D). Several skews, and especially the purine skew (Figure 4E) indicate that the SARS-coronavirus genome is the most variable with regard to consistency of nucleotide composition across the genome. This is consistent with the ancient recombination events among ancestral coronaviruses proposed by Zhang *et al*. [29] after employing a battery of BLAST searches and 7 recombination detection techniques. Others have also examined nucleotide skews of coronaviruses, but this has been limited to GC skews [30,31] that show a depletion of cytosine relative to guanosine in the 10 Kb at the 3' end of the SARS-coronavirus genome. Both Grigoriev [30] and Pyrc *et al*. [31] suggested that this resulted from the unusual transcription process of the coronaviruses, but differed in their detailed models.

## Conclusion

GraphDNA is an easy to use Java Web Start application designed to display and compare DNA sequences graphically using three available methods – DNA walks, cumulative purine/keto skews and cumulative dinucleotide skews. Although others have used nucleotide skews to analyze genomes and have predicted coding strand selection, replication origin and termini sites [32,33], the tools to perform these analyses have not been readily accessible by the general research community. In contrast, GraphDNA is freely accessible via the Internet and runs on most computer platforms; in addition the source code is available through an Open Source license. GraphDNA offers a quick and easy method to compare the nucleotide skews of genomes and provides an additional analysis tool that is especially useful for characterization of distantly related nucleotide sequences.

## Availability and requirements

**Project Name: **GraphDNA

**Project Home Page: **GraphDNA may be accessed from the workbench at 

**Operating Systems: **All platforms supporting Sun's JRE version 1.4.1 or compatible

**Programming Languages: **Java, SQL

**Other requirements: **Java 1.4 or higher

**License: **Open Software License 

## Authors' contributions

JMT designed the interface and coded the prototype; DH and GB coded the final application; VT and CU described and specified the features of GraphDNA, tested the application, provided usage examples and wrote the manuscript. All authors read and approved the final manuscript.
